# Protective Effects of Nettle Tea on SKOV-3 Ovarian Cancer Cells Through ROS Production, Apoptosis Induction, and Motility Inhibition Without Altering Autophagy

**DOI:** 10.3390/foods13203336

**Published:** 2024-10-21

**Authors:** Maria Abi Akl, Roy Hajj, Georgio Jamati, Louna Karam, José-Noel Ibrahim, Philippe H. Kobeissy, Maria Younes, Sandra Rizk

**Affiliations:** 1 Department of Biological Sciences, Lebanese American University, Byblos P.O. Box 36, Lebanon; maria.abiakl01@lau.edu (M.A.A.); roy.elhajj01@lau.edu (R.H.); louna.karam@lau.edu.lb (L.K.); josenoel.ibrahim@lau.edu.lb (J.-N.I.); philippehussein.kobeissy@lau.edu.lb (P.H.K.); 2 Department of Biology, Texas A&M University, College Station, TX 77843, USA; gioj@tamu.edu

**Keywords:** *Urtica dioica*, ovarian cancer, apoptosis, alternative medicine, autophagy, cell motility

## Abstract

*Urtica dioica* L. (UD), also known as the stinging nettle, has long been used in traditional medicine for its wide range of health benefits. The current study focuses on the effect of nettle tea on the growth and proliferation of one of the most aggressive ovarian adenocarcinoma cell line, SKOV-3 cells. To examine this, cytotoxicity, cell cycle analysis, and ROS assays were performed, along with Annexin V/PI dual staining, cell death ELISA, Western blot analysis, and motility assays. The results showed that a UD aqueous extract (UDAE) can inhibit the growth and proliferation of SKOV-3 cells in a dose- and time-dependent manner by promoting cellular fragmentation. This was accompanied by an increase in two apoptotic hallmarks, the flipping of phosphatidylserine to the outer membrane leaflet and DNA fragmentation as revealed by cell death ELISA. This aqueous extract showed a pro-oxidant activity while also activating the extrinsic caspase-dependent apoptotic pathway with no alteration in autophagy markers. Furthermore, the extract showed promising inhibitory effect on the migratory capacities of aggressive ovarian cancer cells, in vitro.

## 1. Introduction

Accounting for 4% of all cancer cases in women, ovarian cancer is the most common cause of death among gynecologic malignancies and the fifth leading cause of cancer-related death following lung, breast, colorectal, and pancreatic cancers [1,2,3]. It is histologically classified according to its origin from epithelial, germ, and mesenchymal cells (the sex cord and stroma), with epithelial ovarian tumors accounting for the majority of cases [4]. Because ovarian cancer in its early stages is usually asymptomatic or presents with nonspecific pelvic or abdominal symptoms, more than 70% of ovarian cancer cases go undiagnosed and undetected until the disease has reached stages III or IV with involvement of the peritoneal cavity and other organs [1,2,3,5]. Current treatment involves cytoreductive surgery (surgical debulking) followed by combination chemotherapy with platinum compounds [1,2]. The prognosis strongly depends on the stage and grade of the disease, and because most cases are diagnosed at advanced stages, ovarian cancer is generally associated with a poor prognosis [1,3]. One of the most widely used and aggressive ovarian cancer cells is the SKOV-3 cell line derived from a 64-year-old female with ovarian adenocarcinoma [6]. This cell line is often referred to as high-grade serous ovarian carcinoma (HGSOC) for its aggressive behavior and ability to metastasize throughout the peritoneal cavity [7]. In a study conducted by Hallas-Potts et al., eleven different ovarian cancer cell lines were compared to assess proliferative and invasive abilities showing that SKOV-3 cells are one of the most aggressive ovarian cancer cell lines given their highly invasive capabilities [8].

Despite the increased effectiveness and better survival rates provided by chemotherapy, its side effects and long-term consequences still constitute a substantial concern for patients and healthcare providers alike [9]. This highlights the need for approaches to alleviate the side effects for patients undergoing chemotherapy, with plant-based treatments holding promising potential in that regard. In fact, natural products have gained attention for their role in preventing and enhancing chemotherapy for ovarian cancer. Naturally derived compounds such as quercetin, curcumin, lycopene, and tannins, among several other compounds, exhibit promising therapeutic potential by promoting apoptosis, DNA damage, and inhibiting angiogenesis in various ovarian cancer cell lines.

*Urtica dioica* L. (UD), commonly known as the stinging nettle, is a perennial herbaceous flowering and edible plant known to possess nutritional and medicinal properties [10]. It belongs to the Urticaceae family and grows in temperate wasteland and tropical areas worldwide [11]. As suggested by its name, this plant has stinging hairs found on its stems and leaves, which cause dermatitis when in contact with the skin [12,13]. However, cooking or steaming before consumption are effective methods of destroying these stinging hairs [14]. UD is known to be rich in flavonoids, tannins, isolectins, sterols, terpenes, fatty acids, polysaccharides, protein, vitamins, and minerals [10,15]. Some of these bioactive compounds, including monounsaturated and polyunsaturated fatty acids, lectins, sterols, polysaccharides, and lignans, have been found to inhibit angiogenesis as well as the proliferation and migration of different types of cancers, including glioblastoma and lung cancer [12,16]. In addition, different flavonoids found in the nettle plant such as quercetin, apigenin and luteolin were found to exert pro-oxidant and pro-apoptotic activity via DNA damage, cell cycle arrest, and PARP and caspase cleavage in liver and colorectal cancer [17,18]. Among the bioactive constituents are the ferulic acid derivatives previously reported for their anticancer activity on breast, colon, liver, and lung cancer by promoting ROS accumulation, cell cycle arrest, the activation of the intrinsic apoptotic pathway, and the inhibition of migration, invasion, and angiogenesis [19]. Studies have demonstrated the effectiveness of plant-based infusions, historically known for their potential effect in preventing and treating cancer [20]. The nettle plant is known to possess a wide range of health benefits, including chemopreventive properties against leukemia, breast, prostate, and colon cancers, as well as analgesic, antimicrobial, antidiabetic, anti-inflammatory, antioxidant, antiaging, and antiproliferative effects and systemic protective properties for the liver, kidneys, and cardiovascular, digestive, reproductive, and nervous systems [10,12,21]. Evaluating the toxicity and safety levels of UD was of great interest as it has been widely used as an alternative or complementary strategy for the treatment of cancer or other diseases. The nettle plant demonstrated a high margin of safety in vivo [22] and biocompatibility in humans [12], suggesting promising and safe implementation in clinical strategies to improve patients’ health.

The widespread health benefits of UD have been documented in the literature, but evidence of its anticancer properties are still limited, with studies restricted to breast, lung, gastrointestinal, prostate, and leukemic cancer cells [23,24,25,26,27]. The literature suggests that one of the most important mechanisms by which UD exerts its anti-cancer effects is via the apoptotic pathway by promoting DNA fragmentation and phosphatidylserine translocation to the outer membrane leaflet. Extensive studies reported the promising potential of UD to activate apoptosis via the intrinsic or extrinsic pathways in prostate, lung, and breast cancer, among others, as detailed by Esposito et al. [11].

Several studies have been performed to evaluate the effects of natural plant-derived compounds on ovarian cancer. In fact, more than 34 natural compounds have been studied with respect to ovarian cancer [28]. However, to our knowledge, no previous studies have investigated the anticancer effect of UD on ovarian cancer, highlighting the need to explore this type, particularly using SKOV-3 cells, which have been established as one of the most aggressive cell lines (detailed above). As such, this paper aims to fill this gap by examining the possibility that an aqueous extract of UD exhibits antiproliferative and chemo-preventive properties against SKOV-3 cells in vitro. Specifically, we aim to determine whether this extract reduces cellular viability by promoting the activation of programmed cell death mechanisms, such as apoptosis or autophagy, and inhibiting cellular motility, thus offering a promising therapeutic value for ovarian cancer patients.

## 2. Methods

### 2.1. UD Aqueous Extract Preparation

UD leaves were collected, identified, and characterized as previously described by Hodroj et al. [27]. Briefly, the leaves were allowed to dry at room temperature, and an aqueous extract was prepared in type I autoclaved boiling water for 20 min. The extract (5% *w*/*v*) was decanted, strained through a cheesecloth, and then filtered using a syringe filter with a 0.45 µm pore size. The extract was then divided into aliquots that were stored at −20 °C. On the day of treatment, the aliquots were thawed and applied to ovarian cancer cells using the appropriate concentrations.

### 2.2. Cell Culture

SKOV-3 ovarian cancer cells were obtained from the American Type Culture Collection (ATCC) and cultured in Dulbecco’s Modified Eagle Medium (DMEM media (Sigma-Aldrich, St. Louis, MO, USA) supplemented with 10% Fetal Bovine Serum (FBS, Gibco™, Dublin, Ireland) and 1% antibiotics (100 U/mL penicillin and 100 µg/mL streptomycin from Pen-Strep Lonza, Basel, Switzerland) [29]. The cells were placed in a humidified incubator at 37 °C and 5% CO_2_ and were checked daily using the ZOE fluorescent cell imager (BioRad, Hercules, CA, USA). The cells were split three times per week and viability was assessed regularly using trypan blue and a hemocytometer.

### 2.3. MTS Cytotoxicity Assay

The viability of SKOV-3 cells was measured using the MTS (Promega, Fitchburg, WI, USA) cell viability reagent. Cells were cultured in 96-well plates in triplicate at a confluency of 5 × 10^4^ cells/mL. After overnight incubation, the cells were treated with media for the control and with increasing concentrations of UDAE (6, 8, 10, 12, 15% *v*/*v*). After 24, 48, or 72 h of treatment, MTS reagent was prepared and added to quantify cell viability [30]. The conversion of tetrazolium salt to formazan by metabolically active cells was detected by a color change measured using spectrophotometry (Varioskan™ LUX, Thermo Scientific, Waltham, MA, USA) at a wavelength of 492 nm [31]. Percent proliferation was calculated by dividing the absorbance of the treated cells with the average absorbance of the untreated control cells. The half-maximal inhibitory concentration (IC_50_) was calculated using Graphpad Prism version 9.1.0 through a non-linear regression curve of a dose–response variable slope.

### 2.4. Cell Cycle Analysis

Cells were seeded in 6-well plates at a confluency of 1 × 10^5^ cells/mL. The following day, cells were treated with increasing concentrations of UDAE (8, 10, 12, 15% *v*/*v*), and cisplatin (10 μM) was used as a positive control for 24 and 48 h. Then, cells were collected, fixed with ice-cold PBS and ethanol on ice, and stored at −80 °C overnight. Fixed cells were then stained with Propidium iodide (50 μg/mL) (PI) (Abcam, Cambridge, UK) and 0.5 μg/mL RNase (Roche, Basel, Switzerland) for 45 min in the dark. The DNA content was studied using the Guava easy Cyte™ flow cytometer (Merck Millipore, Darmstadt, Germany), and accordingly, cells were classified into the various phases of the cell cycle: sub-G0/G1 (Pre-G cells) having <2n content, G0/G1 having 2n, S having between 2n and 4n, and G2/M having 4n DNA content.

### 2.5. Reactive Oxygen Species (ROS) Assay

The DCFDA cellular ROS detection assay kit (Abcam, Cambridge, UK) was used to assess the instant intracellular production of ROS. SKOV-3 cells were seeded at a density of 1 × 10^5^ cells/mL in 96-well plates. The following day, they were pre-treated with H_2_DCFDA followed by the addition of treatment for 2 h of UDAE (8, 10, 12, 15% *v*/*v*) or H_2_O_2_ (10 μM) used as a potent ROS inducer. As previously detailed by Haykal et al., the presence of ROS promotes the conversion of H_2_DCFDA into the highly fluorescent 2′,7′-dichlorofluorescein (DCF) molecule, which is quantified by spectrofluorometry using the Varioskan™ LUX multimode microplate reader (Thermo Fisher Scientific, Bremen, Germany) [32].

### 2.6. Annexin V/PI Dual Staining

SKOV-3 cells were seeded in 6-well plates at a confluency of 1 × 10^5^ cells/mL. After overnight incubation, cells were treated with increasing concentration of UDAE (8, 10, 12, 15% *v*/*v*), with negative controls being only treated with DMEM media, and positive controls with cisplatin (10 μM). After 24 and 48 h of treatment, cells were collected as previously described by Nafeh et al. [29]. Cells were stained with Annexin V and PI (Annexin V-FITC Apoptosis Staining Kit, Abcam Inc., Cambridge, UK), to be later analyzed using the Guava easy Cyte™ flow cytometer. During apoptosis, phosphatidylserine is flipped from the inner membrane to the outer one, giving positive staining for Annexin, while a positive staining for PI is obtained upon loss of cell membrane integrity and its binding to the nucleic acid content. Cells were grouped as follows: Annexin^−^, PI^−^ (viable cells; lower left quadrant), Annexin^+^, PI^+/−^ (apoptotic cells; right quadrants), and Annexin^−^, PI^+^ (necrotic; upper left quadrant), as reported in the results.

### 2.7. Cell Death ELISA

Cells were seeded in 6-well plates at a concentration of 1 × 10^5^ cells/mL and incubated overnight, after which they were treated with increasing concentrations of UDAE (8, 10, 12% *v*/*v*) and cisplatin (10 μM) for 48 h. After the desired incubation period, cells were collected and lysed according to the manufacturer’s instruction and as previously described by El Khoury et al. [33]. Cell lysate was incubated in a microplate pre-coated with anti-histone antibodies, followed by the addition of anti-DNA antibody conjugated to peroxidase enzyme. Using the Varioskan™ LUX (Thermo Scientific, Waltham, MA, USA) spectrophotometer plate reader, DNA fragmentation was quantified after the addition of the colorimetric ABTS substrate. The enrichment for oligo-nucleosomal fragments was calculated by dividing the absorbance of the treated sample by the absorbance of the control sample.

### 2.8. Reverse-Transcription Quantitative Real-Time PCR (RT-qPCR)

SKOV-3 cells were plated at a density of 5 × 10^4^ cells/mL in 6-well plates and left untreated or treated with 5%, 10%, or 15% UDAE. Following 24 h of treatment, total RNA was extracted using the RNeasy Plus Mini kit (Qiagen, Düsseldorf, Germany) and 1 µg of RNA was reverse transcribed using the iScript cDNA Synthesis Kit (BioRad, Hercules, CA, USA) according to the manufacturer’s instructions. qRT-PCR was performed using the iTaq Universal SYBR Green Supermix (BioRad, Hercules, CA, USA) as previously described [34]. Samples were analyzed in triplicate and the relative expressions of cyclins A1, A2, B1, B2, D1, D2, E1, and E2 were calculated by the 2^−ΔΔCt^ method using the housekeeping gene *GAPDH* as a reference gene. The primers (TIB Molbiol, Berlin, Germany) used are reported in the Supplementary Materials (Supplementary Table S1).

### 2.9. Western Blot

To evaluate the effect of UDAE at a molecular level, protein expression of regulatory markers was performed by Western blot analysis. As such, SKOV-3 cells were seeded and incubated overnight in 6-well plates at a confluency of 1 × 10^5^ cells/mL. Cells were treated with 10 and 12% *v*/*v* UD for 48 h, after which the cells were lysed using the RIPA buffer and the protease inhibitor cocktail which consists of AEBSF, Sodium EDTA, Leupeptin, and Pepstain A (MP Biomedical, Santa Ana, CA, USA) [35]. Extracted proteins were quantified using the Lowry method, separated by SDS-PAGE, and transferred to PVDF membranes, which were blocked with 5% skimmed milk and incubated overnight with primary antibodies of interest: anti-β-actin (Santa Cruz, Dallas, TX, USA), anti-Bax (Invitrogen, Waltham, MA, USA), anti-Bcl2 (Invitrogen, Waltham, MA, USA), anti-cleaved PARP (Invitrogen, Waltham, MA, USA), anti-cleaved-caspase-3 (Invitrogen, Waltham, MA, USA), anti-cleaved-caspase 8 (Cell Signaling, Danvers, MA, USA), anti-Beclin (Invitrogen, Waltham, MA, USA), and anti-LC3B (Cell Signaling, Danvers, MA, USA). The following day, the membranes were washed and incubated with the appropriate secondary antibodies (BioRad, Hercules, CA, USA) at room temperature for 1 h. After another wash, Clarity™ Western ECL substrate (BioRad, Hercules, CA, USA) was used to obtain images on the ChemiDoc machine (BioRad, Hercules, CA, USA) [36]. ImageJ software (version 1.52a) was used to quantify blot bands and calculate the relative expression of proteins.

### 2.10. MTS Cytotoxicity Assay with ZVAD

SKOV-3 cells were cultured in 96-well plates in triplicate at a confluency of 5 × 10^4^ cells/mL. After overnight incubation, the cells were pre-treated with ZVAD (50 μM), a caspase inhibitor, for 30 min prior to the addition of UDAE (8, 10, 12% *v*/*v*) for 48 h. MTS viability reagent was used as previously described to quantify metabolically active cells and assess the caspase-dependent cytotoxic effect of the treatment. The Varioskan™ LUX plate reader was used to quantify the change in color at a wavelength of 492 nm [37].

### 2.11. Wound Healing Assay

To evaluate the inhibitory effect of UDAE on the migration properties of ovarian cancer cells, a wound healing assay was performed. SKOV-3 cells were seeded in 24-well plates at a density of 1 × 10^5^ cells/mL and allowed to reach a confluent monolayer. The following day, a scratch was made in each well prior to the addition of UDAE (8, 10, 12% *v*/*v*) for 48 h. The concentrations used were chosen carefully to make sure the results obtained are due to the inhibitory effect of the extract rather than its cytotoxic potential. Images were taken after the scratch was made at T = 0, as well as after 48 h of treatment (T = 48 h), using the ZOE fluorescent cell imager. Quantification was performed using the ImageJ program (version 1.52a), allowing the measurement of the extent to which the treatment prevented the closure of the scratch.

### 2.12. Migration Assay

To further confirm the ability of UD to inhibit the migration of ovarian cancer cells, trans-membrane migration assay was performed. SKOV-3 cells were cultured and starved in serum-free media supplemented with 0.5% FBS overnight. The following day, cells were seeded in porous membrane chambers (8 μm pore size) at a density of 3 × 10^5^ cells/mL in serum-free medium. Mild concentrations of UDAE (8, 10 and 12% *v*/*v*) were added for 48 h to the inserts to make sure the results obtained are due to its inhibitory effect rather than its cytotoxicity. The inserts were then placed in 24-well plates containing complete media (with 10% FBS). After the desired incubation period, the inserts were washed using PBS and cotton swabs were used to gently swab the inside of the inserts. Formaldehyde and methanol were then added to the inserts to fix and permeabilize the cells, respectively. Cells were then stained using DAPI solution (1 μg/mL) and visualized using the ZOE fluorescent cell imager. The ImageJ program (version 1.52a) was used to quantify the images taken from different fields and determine the number of migratory cells across the membrane.

### 2.13. Statistical Analysis

All experiments were performed in triplicates and repeated three independent times. Excel was used for the calculations while GraphPad Prism Version 9.1.0 was used to determine the statistical significance by one-way or two-way ANOVA according to the experiment. The results were reported as mean ± SD. Statistically significant differences were reported with * indicating *p*  <  0.5, ** indicating *p*  <  0.05, and *** indicating *p*  <  0.001.

## 3. Results

### 3.1. Nettle Tea Exhibits an Antiproliferative Effect on SKOV-3 Ovarian Cancer Cells

**Figure 1:**
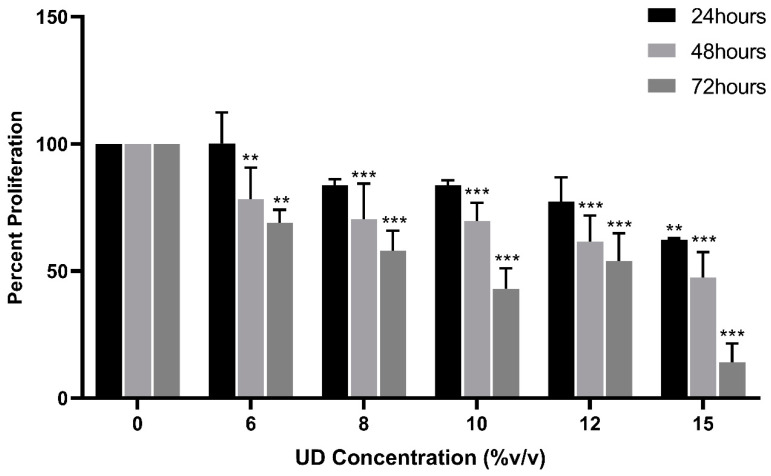
MTS cell viability assay of SKOV-3 cells treated with increasing concentrations of UD (6, 8, 10, 12, and 15% *v*/*v*) for 24, 48, and 72 h. Results are compared to the untreated cells and the data are represented as mean ± SD from three separate experiments. Statistical significance is reported as ** to *p*-value < 0.05, and *** to *p*-value < 0.001.

The MTS cell viability assay was performed to quantify the effect of UDAE on cell proliferation. SKOV-3 cells were cultured and treated with increasing concentrations of UD for 24, 48, and 72 h. A significant dose- and time-dependent decrease in cell viability was observed, with cell proliferation being reduced to 62% at 24 h, 47% at 48 h, and 14% at 72 h at the highest concentration of UD (15% *v*/*v*) (Figure 1). The IC_50_ values were determined to be 18.5, 16, and 9.3% *v*/*v*, respectively for 24, 48, and 72 h. These results show that the aqueous extract of nettle plant inhibits the proliferation of SKOV-3 ovarian cancer cells in a dose- and time-dependent manner.

### 3.2. Nettle Tea Induces Cellular Fragmentation in SKOV-3 Ovarian Cancer Cells

**Figure 2:**
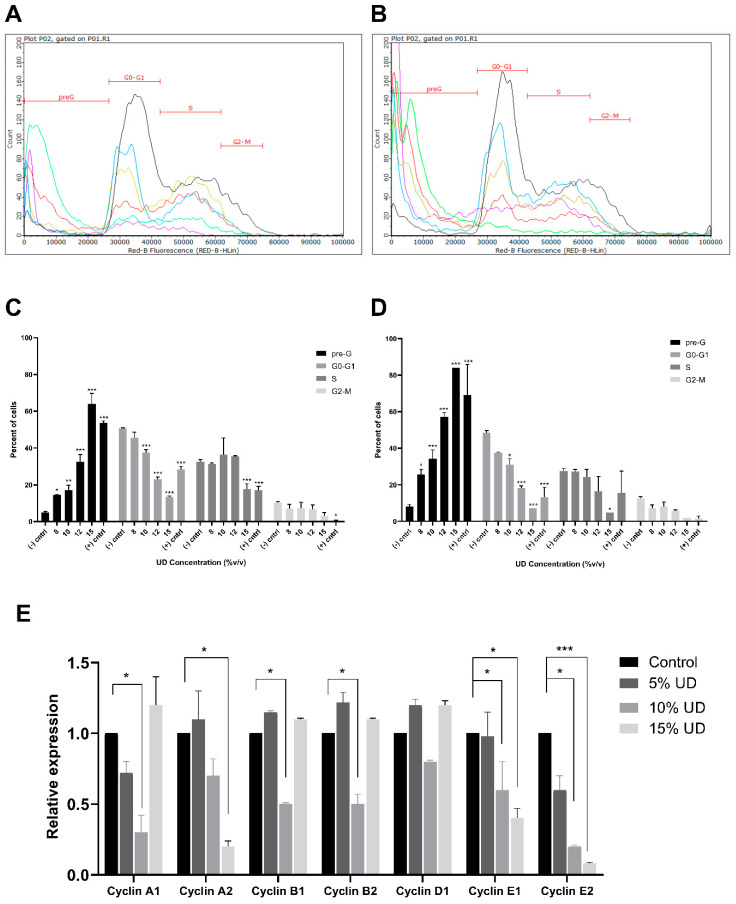
Overlay plots of cell cycle analysis for SKOV-3 cells treated with increasing concentrations of UD (8, 10, 12, and 15% *v*/*v*) for 24 (**A**,**C**) and 48 h (**B**,**D**). In the overlay plots, black, blue, yellow, red, green, and purple represent control (-cntrl), 8, 10, 12, 15% *v*/*v* UD and cisplatin, respectively. Relative expression of cyclins A1, A2, B1, B2, D1, E1, and E2 by RT-qPCR in SKOV-3 cells treated with increasing concentrations (5%, 10%, and 15% *v*/*v*) of UD for 24 h. *GAPDH* was used as a reference gene (**E**). Results are compared to the untreated cells and the data are represented as mean ± SD from three separate experiments. Statistical significance is reported as * corresponding to *p*-value < 0.5, ** to *p*-value < 0.05, and *** to *p*-value < 0.001.

Cell cycle analysis was performed to assess whether cell cycle arrest is responsible of the cytotoxic effect observed in ovarian cancer cells. As such, cells were stained with PI and cell cycle distribution based on the DNA content was performed using flow cytometry. The results showed a dose- and time-dependent shift in SKOV-3 cells from the G0/G1, S, and G2/M phases to the pre-G phase, indicating cellular fragmentation upon exposure of the cells to the extract for 24 and 48 h (Figure 2A,B). There was a significant increase in the pre-G cells, going from 5% to 64% at 24 h with the highest UD concentration (15% *v*/*v*), along with a significant decrease from 50% to 13%, 32% to 17%, and 10% to 3% at the G0-G1, S, and G2-M phases, respectively, as compared to the control (Figure 2C). Similarly, cells treated with 15% *v*/*v* UD for 48 h showed a significant increase from 8% to 84% at the pre-G phase along with a significant decrease from 48% to 7%, 27% to 4%, and 12% to 2% as compared to the control, at the G0-G1, S, G2-M phases, respectively (Figure 2D). These findings suggest that the nettle tea preparation halts the cellular proliferation of SKOV-3 by promoting cellular fragmentation rather than inducing cell cycle arrest. The data obtained from RT-PCR reveal no increase in any of the cyclins investigated, which further corroborates these findings (Figure 2E).

### 3.3. Nettle Tea Enhances ROS Production in SKOV-3 Ovarian Cancer Cells

**Figure 3:**
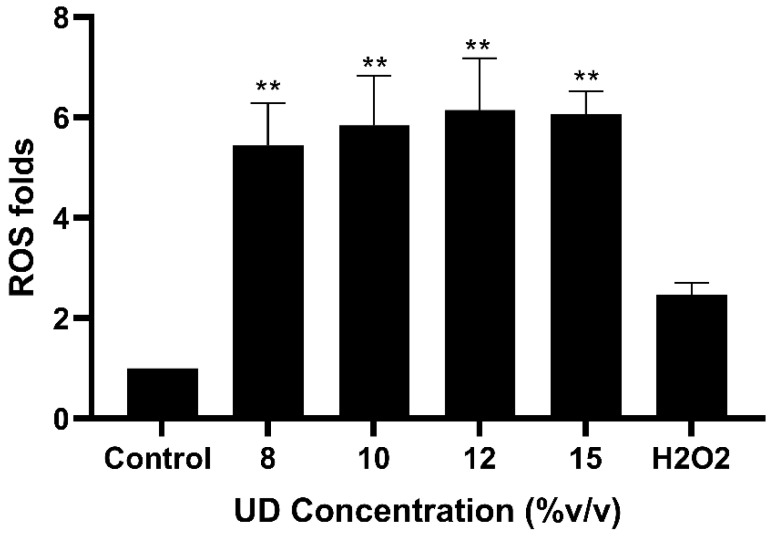
Reactive oxygen species assay in SKOV-3 cells treated with increasing concentrations of UD (8, 10, 12, and 15% *v*/*v*) or H_2_O_2_ (10 μM) used as a potent ROS inducer. Results are compared to the untreated cells and the data are represented as mean ± SD from three separate experiments. Statistical significance is reported as ** to *p*-value < 0.05.

We aimed at evaluating the levels of ROS given the cytotoxic effect of the UDAE observed previously on ovarian cancer cells. DCFDA assay was performed to quantify the instant production of ROS in SKOV-3 cells treated with UD showing a significant dose-dependent increase in ROS production, with a six-fold increase in cells treated with 15% *v*/*v* UD as compared to the control (Figure 3). Our results were compared to the potent ROS inducer H_2_O_2_ and were found to be noticeably higher in SKOV-3 cells. These results suggest that nettle tea exhibits a pro-oxidant effect on ovarian cancer cells in vitro.

### 3.4. Nettle Tea Promotes Apoptosis in SKOV-3 Ovarian Cancer Cells

**Figure 4:**
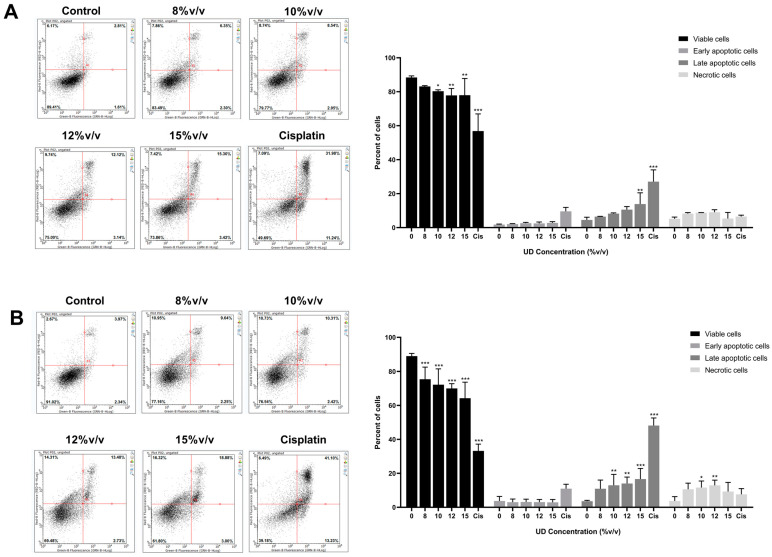
Annexin V/PI dual staining in SKOV-3 cells treated with increasing concentrations of UD (8, 10, 12, and 15% *v*/*v*) for 24 (**A**) and 48 h (**B**). Cisplatin (Cis) was used as a positive control. Dot plot images representing different populations: bottom-left quadrant representing living cells, bottom- and top-right quadrants representing early and late apoptotic cells, respectively, and top-left quadrant representing necrotic cells. Results are compared to the untreated cells and the data are represented as mean ± SD from three separate experiments. Statistical significance is reported as * corresponding to *p*-value < 0.5, ** to *p*-value < 0.05, and *** to *p*-value < 0.001.

Since ROS are known to be interconnected to various modes of cell death mechanisms, we aimed to investigate the role of UD in promoting apoptosis. One hallmark of apoptosis is phosphatidyl serine translocation from the inner to the outer leaflet. As such, Annexin V/PI dual staining was performed on SKOV-3 cells treated with various concentrations of UD for 24 and 48 h. The results showed a significant decrease in the percentage of viable cells (Ann^−^/PI^−^) and an increase in apoptotic cells (Ann^+^/PI^+/−^) (Figure 4). After 24 h of treatment with the highest concentration of UD (15% *v*/*v*), the percentage of viable cells significantly decreased from 88% to 78%, while the percentage of apoptotic cells increased from 5% to 15% (Figure 4A). A similar pattern was observed upon treatment for 48 h, with the percentages of viable cells reaching 65% and apoptotic cells 20% compared to the control (Figure 4B). These results demonstrate the promising effect of the aqueous extract of nettle plant on promoting apoptosis in ovarian cancer cells.

**Figure 5:**
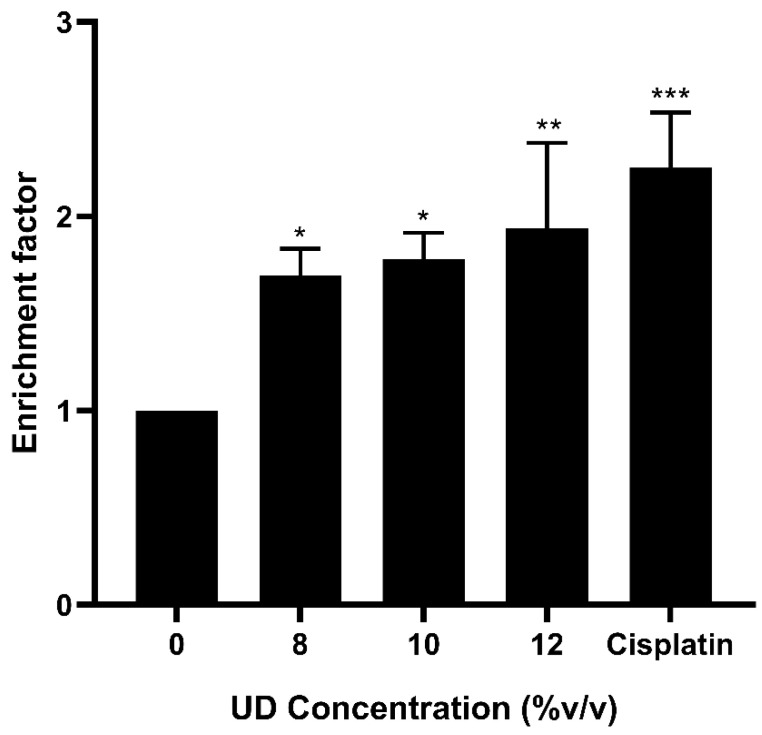
DNA fragmentation detection via cell death ELISA in SKOV-3 cells upon treatment with UD (8, 10, 12% *v*/*v*) for 48 h. Results are compared to the untreated cells and the data are represented as mean ± SD from three separate experiments. Statistical significance is reported as * corresponding to *p*-value < 0.5, ** to *p*-value < 0.05, and *** to *p*-value < 0.001.

Given the positive results obtained, we aimed at investigating another hallmark of apoptosis, which is nuclear DNA fragmentation. Cell death detection ELISA was performed on SKOV-3 cells treated with increasing doses of UD for 48 h. Our results revealed a significant dose-dependent increase in the enrichment for oligo-nucleosomal fragments, with an approximate two-fold increase at the highest concentration (12% *v*/*v*), consistent with the effect obtained using the positive control (Figure 5). This significant increase in controlled DNA fragmentation further confirms the activation of apoptosis in SKOV-3 cells upon exposure to UDAE.

**Figure 6:**
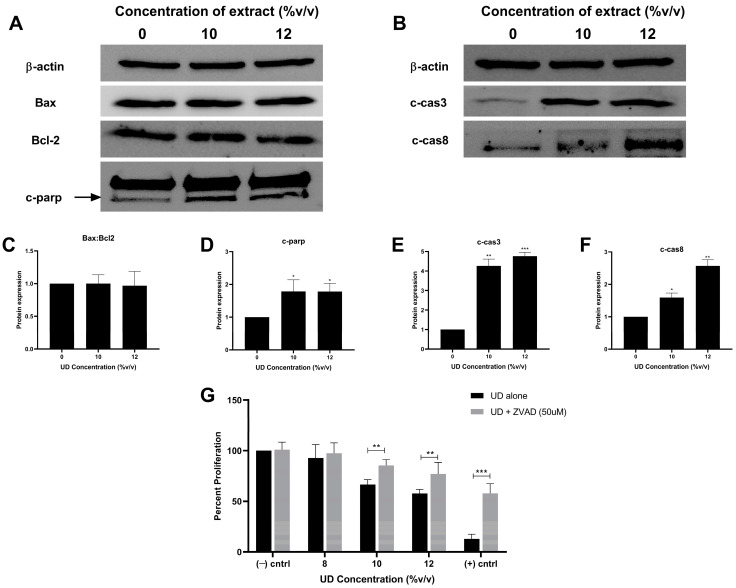
Western blot analysis of the intrinsic (**A**) and extrinsic (**B**) regulatory proteins in SKOV-3 cells treated with different concentrations of UD (10 and 12% *v*/*v*) for 48 h. Protein expression of Bax and Bcl2 ratio (Bax:Bcl2) (**C**), cleaved-PARP (c-PARP) (**D**), cleaved-caspase 3 (c-cas3) (**E**), and cleaved-caspase 8 (c-cas8) (**F**) was quantified using Image J. MTS cell viability assay was performed following UD treatment alone or in combination with the caspase inhibitor ZVAD for 48 h in SKOV-3 cells. Cisplatin was used as a positive control ((+) cntrl) (**G**). Results are compared to the untreated control cells (−) cntrl and the data are represented as mean ± SD from three separate experiments. Statistical significance is reported as * corresponding to *p*-value < 0.5, ** to *p*-value < 0.05, and *** to *p*-value < 0.001.

To decipher the molecular pathway underlying the upregulation of apoptotic hallmarks upon UD exposure, Western blot analysis of apoptotic markers was performed (Figure 6A–F). The expression of the mitochondrial regulatory proteins, namely, the pro-apoptotic Bax (Bcl-2 associated X protein) and anti-apoptotic Bcl-2 (B-cell-lymphoma protein 2) proteins, remained unchanged upon treatment with the extract, and hence no alteration in their ratio was detected (Figure 6C). However, the cleaved form of the DNA repair protein PARP (Poly (ADP-ribose) polymerase) was found to be significantly upregulated, reaching approximately a two-fold increase upon treatment with UD, further indicating DNA fragmentation. The cleaved caspase-8, which is involved in the extrinsic pathway of apoptosis, was evaluated and found to be significantly expressed reaching a 2.5-fold increase at the highest concentration of treatment. To confirm the activation of the execution pathway, which is downstream of both intrinsic and extrinsic apoptotic pathways, caspase-3 cleavage was then assessed; a significant increase in the cleaved caspase-3 protein was observed reaching a four-fold increase at the 12% (*v*/*v*) concentration of UD. These results support our previous findings and further reveal the specific apoptotic pathways activated upon treatment with nettle tea, namely the extrinsic pathway.

To further confirm the caspase-dependent cell death mechanism activated by UDAE, a cytotoxicity assay was performed on SKOV-3 cells treated with the extract alone or in combination with the caspase inhibitor ZVAD. A significantly higher dose-dependent percent proliferation was observed upon pre-treatment with ZVAD as compared to the cells treated with only UD; the percent proliferation of cells treated with the highest concentration of UD was reported to be 58%, as compared to 77% in the cells treated with ZVAD + UD (Figure 6G). These results show that ZVAD was able to revert the cytotoxic effect observed with the extract alone, confirming the activation of apoptosis in a caspase-dependent manner.

### 3.5. Nettle Tea Effect on Autophagy in SKOV-3 Ovarian Cancer Cells

**Figure 7:**
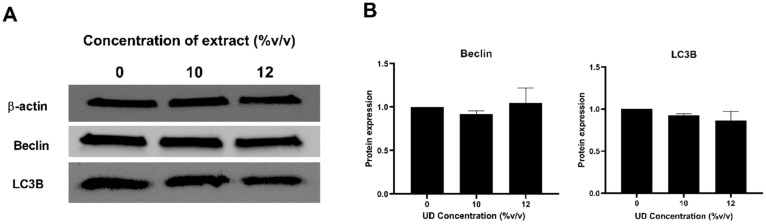
Western blot analysis of autophagy related markers in SKOV-3 cells treated with different concentrations of UD (10 and 12% *v*/*v*) for 48 h (**A**). Protein expression of Beclin and LC3B was quantified using Image J software (**B**). Results are compared to the untreated cells and the data are represented as mean ± SD from three separate experiments.

Since previous studies have reported correlations between ROS production and autophagic cell death mechanisms, we aimed to investigate whether UDAE can promote the activation of autophagy in ovarian cancer cells. Western blot analysis was performed to analyze key regulatory proteins that govern the first and last steps of autophagy, particularly Beclin and LC3B (microtubule-associated protein 1 light chain 3 beta) proteins, respectively (Figure 7A). Our results demonstrate that the aqueous UD extract does not alter the expression of autophagy-related markers as revealed by their constant expression when compared to the control (Figure 7B). These results suggest that apoptosis is the mechanism responsible of the cell death induced by UDAE rather than autophagy.

### 3.6. Nettle Tea Inhibits the Motility of SKOV-3 Ovarian Cancer Cells

**Figure 8:**
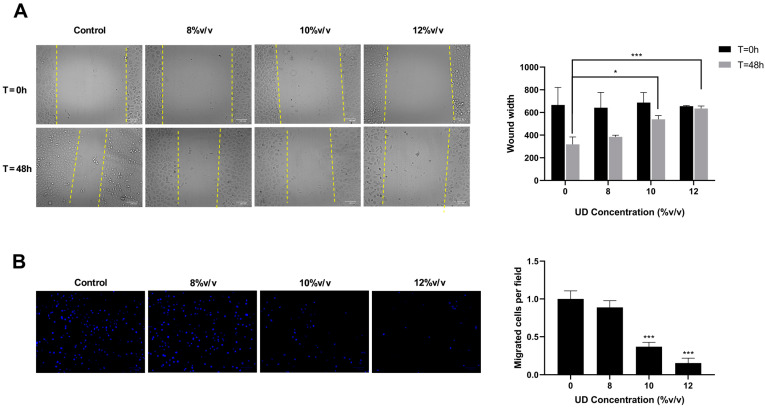
Wound healing (**A**) and trans-membrane migration (**B**) assays performed and quantified using Image J software on SKOV-3 cells treated with 8, 10, and 12 % *v*/*v* UD for 48 h. Results are compared to the untreated cells and the data are represented as mean ± SD from three separate experiments. Statistical significance is reported as * corresponding to *p*-value < 0.5 and *** to *p*-value < 0.001.

The effect of UDAE on the motility of SKOV-3 cells was evaluated by wound healing and trans-membrane migration assays. In the wound healing assay, images taken after 48 h of the treatment (at T = 48 h) showed a noticeable decrease in the wound closure while increasing the concentrations of UD (Figure 8A). After quantification of the results, there was a significant dose-dependent increase in the wound width, which doubled as compared to the control at the highest concentration (12% *v*/*v*). Our results revealed almost no change in the wound width at T = 48 h as compared to T = 0 h, confirming the inhibitory effect of this extract on the motility of SKOV-3 cells relative to the control.

In the migration assay, a significant decrease in the number of cells that moved across the porous membrane was observed (Figure 8B). Upon quantification of the migrated cells per field and normalization to the control, the ratio obtained for the cells treated with the highest concentration of UD reached 0.1. These findings further support the promising effect of UDAE on inhibiting the motility and migration of SKOV-3 ovarian cancer cells.

## 4. Discussion

Medicinal plants represent a rich and diverse source of chemical compounds used for centuries in the treatment of various forms of ailments, including cancer [38]. *Urtica dioica*, commonly known as stinging nettle, is a well-known medicinal plant having long been used in traditional medicine for its various health benefits, with a range of potential applications such as dietary supplements, functional food, and pharmacological formulations [12,39]. Particularly, the anticancer properties of UD aqueous extracts have been a focus for our laboratory, where we have previously demonstrated its antitumor potential on acute myeloid leukemia (AML) cells, in addition to its ability to sensitize triple-negative breast cancer (TNBC) cells to cisplatin treatment [27,29]. Since no previous studies have reported the potential effect of UD in ovarian cancer, we aimed to investigate the chemopreventive effect of an edible, aqueous extract of nettle leaves on the proliferation and migration of the SKOV-3 ovarian adenocarcinoma cell line in vitro. Considering that tea is the second most widely consumed beverage worldwide following water [40], using an aqueous extract holds promising potential as a natural strategy for disease prevention and health maintenance due to its ease of incorporation and integration into the diet. The potential use of UD as a therapeutic approach is demonstrated by its selective effects on cancerous cells while sparing healthy cells. Several studies examining the safety and selectivity of UD on a variety of normal cell lines showed its safety for use given its non-toxic profile.

The data reported here show that nettle tea exhibits a dose- and time-dependent cytotoxic effect on ovarian cancer cells, in line with previously demonstrated antiproliferative effects on several cancer types including breast, prostate, and leukemic cancer cell lines [27,41,42,43]. A study published by Keshavarz et al. reported the potent antiproliferative effect of various UD preparations, such as aqueous, hydroalcoholic, chloroform, and ethyl acetate extracts, on leukemic cells [44]. Furthermore, Kardan et al. showed that a methanolic extract of UD inhibited the growth of the HepG2 hepatocarcinoma and HCT116 colon cancer cell lines [25]. It is noteworthy to highlight that nettle tea was previously reported to possess a minimal effect on the viability of normal cell lines, including human B-lymphocyte (2.4% *v*/*v*) and HFFF2 (human fetal foreskin fibroblast 2) (30–40 ug/mL) cell lines, highlighting its selectivity and safe usage [27,45]. Therefore, UD might be considered a prominent and safe anticancer agent to be used for the treatment of ovarian cancer.

To determine if the antiproliferative effect observed is due to cell cycle arrest, cell cycle analysis was performed. Our results showed that cellular fragmentation was responsible for the antiproliferative effect of UDAE. This is in accordance with previous results by Hodroj et al. showing an increase in cellular fragmentation [27], while in another study UD extract was able to promote a G2/M cell cycle arrest in NSCLC [24] similarly to the effect observed on human colorectal carcinoma HCT116 cells [46]. In contrast, results from Temiz et al. reported a cell cycle arrest at the G0-G1 phase of HL-60 promyelocytic leukemia cells [43]. This shows that the effect of nettle plant on cell-cycle progression is greatly cell-dependent.

We then aimed to assess the effect of UDAE on ROS production to determine whether the extract is promoting oxidative stress in ovarian cancer cells, leading to the cellular damage and fragmentation that was observed. Our results revealed an increase in ROS in SKOV-3 cells treated with UD, similar to previous results reported on human prostate carcinoma LNCaP cells [42]. Another study conducted by Ghasemi et al. also revealed the pro-oxidant activity of UD on colorectal cancer cells through lipid peroxidation and ROS accumulation [47]. Interestingly, high ROS levels are reported to be deleterious to cells by causing oxidative stress, which can trigger cellular damage via the activation of programmed cell death mechanisms [48].

Apoptosis is one of the natural cell death mechanisms that could be triggered by different anticancer agents. Hence, several hallmarks of apoptosis were evaluated, including phosphatidyl serine translocation to the outer membrane and DNA fragmentation [49,50]. The data obtained confirm an increase in the percentage of apoptotic cells, along with an increase in the enrichment for DNA fragments upon treatment with nettle tea. These findings are in accordance with previous results where UD was reported to increase apoptosis and DNA fragmentation in various types of cancer such as leukemia, breast, and prostate cancers [27,42,51]. Previous studies correlated this DNA damage in PC3 and LNCaP prostate cancer cells to the cleavage of PARP protein involved in DNA repair.

To further elucidate the pathway through which UD is promoting apoptosis, key regulatory proteins were evaluated by Western blot analysis. Apoptosis could be triggered via internal or external stimuli promoting the activation of the mitochondrial-dependent or caspase-dependent pathways, respectively. Since ROS accumulation is known to be one of the major internal factors that could promote intrinsic apoptosis by compromising mitochondrial integrity [52,53], we first investigated the proteins that regulate the mitochondrial membrane permeability. Previous studies showed that nettle plants exhibit pro-apoptotic activity on breast cancer cells as revealed by the increase in the Bax:Bcl2 ratio while increasing the mitochondrial membrane permeability and promoting the release of cytochrome c into the cytosol of PC3 prostate cancer cells. Interestingly, the expression of Bax and Bcl2, as well as their ratio, were not altered in SKOV-3 cells treated with nettle tea. Although previous studies have demonstrated the pro-oxidant effect of UD and the activation of the mitochondrial-dependent pathway in various types of cancers [27,42,54,55], the results on ovarian cancer cells suggest that the high levels of ROS detected might not be directly responsible of triggering the intrinsic pathway. Instead, high ROS levels may be a consequence of cellular damage induced by the extract, rather than a mediator of the intrinsic apoptotic pathway. On the other hand, ROS has been reported be involved in the activation of the extrinsic pathway by increasing the expression of the Fas death receptor and downstream effectors [56,57]. Therefore, we then evaluated caspase-8 expression, known to play a pivotal role in the extrinsic pathway, and our results revealed a significant increase in the cleavage of caspase-8 in ovarian cancer cells treated with UDAE. This is in accordance with other studies which demonstrated the pro-apoptotic activity of UD extracts on leukemic and NSCLC cell lines, as summarized by Abi Sleiman et al. and Esposito et al. [11,12]. The auto-proteolytic cleavage of caspase-8 is responsible for promoting downstream effectors of apoptosis, particularly the executioner caspase-3 protein, which was found to be cleaved into its active form in this study [58]. The activation of caspases 8 and 3 mediates the cleavage of several proteins essential for cell survival and function. One of the few recognized cellular substrates of caspases, particularly caspase-3, is Poly [ADP-ribose] polymerase-1 [PARP-1] protein, involved in DNA repair [59]. As revealed in this study, impairment of PARP activity during apoptosis is responsible of the DNA fragmentation observed in ovarian cancer cells treated with the UD aqueous extract [60]. To further validate the activation of the caspase-dependent apoptotic pathway, a cytotoxicity assay was performed using the known caspase inhibitor ZVAD. The results showed that ZVAD reversed the cytotoxic effect observed upon treatment with UD alone, confirming the pivotal role of caspases in promoting death of ovarian cancer cells.

In this study, we were interested in exploring whether autophagy is playing a role in mediating ovarian cancer cell death along with the apoptotic pathway. Therefore, two autophagy-related markers were assessed, Beclin and LC3B proteins. The first one is involved in early stages of autophagy, playing a role as a central regulator interacting with other markers and necessary for autophagosome formation, while LC3B is a marker of later stages of autophagy, being an autophagosome membrane-bound protein [61]. Previous investigations of bioactive compounds found in UD such as quercetin revealed an increase in autophagy in breast, hepatocellular, gastric, and pancreatic cancer cells, while this mechanism was not altered in glioblastoma cells. The data obtained in the current study reveal no variations in autophagy levels in ovarian cancer cells treated with nettle tea, as evident by the constant expression of these two proteins. In fact, it was previously reported that ROS at moderate levels are able to promote the activation of autophagy, whereby, at sufficiently high levels, there is a shift to an apoptotic or necrotic mechanism of death [62]. Taking these results together, we can conclude that UDAE is able to promote high levels of ROS, enough to activate the apoptotic cell death mechanism without altering autophagy levels in ovarian cancer cells.

Knowing that SKOV-3 cells are one of the most aggressive ovarian cancer cells, we aimed at exploring the therapeutic benefits of UDAE on halting the motility and migration of these cells. Migration and wound healing assays were performed on ovarian cancer cells, revealing the promising inhibitory effect of the aqueous nettle plant extract on their migratory capacities. This was demonstrated by the pronounced decrease in the number of migrated cells towards the chemoattractant across the porous membrane, as well as by preventing the closure of the wound in the wound healing assay performed. This is similar to previous results obtained on breast cancer cells, namely MCF-7 and MDA-MB-231, the notably aggressive TNBC cells [45]. Another study highlighted the promising effect of UD in halting cellular motility as revealed via the suppression of miR-21 and other metastasis-related genes in MCF-7 cells.

Medicinal plants have been widely studied for their potential in treating various ailments, including cancer. Their therapeutic value stems from phytochemical compounds like phenols, flavonoids, alkaloids, and terpenoids, which are known for their promising role in the treatment of various diseases [63]. The chemical characterization of nettle tea has previously been performed by our laboratory via LCMS/MS. It was demonstrated that the aqueous UD extract is rich in polyphenols, flavonoids, fatty acids, terpenes, and sesquiterpenes, with three predominant constituents, patuletin, caffeic acid, and m/p-hydroxybenzoic acid [27]. Therefore, it could be hypothesized that the antiproliferative and anticancer properties of UD that have been highlighted in the current study may be attributed to the phytoconstituents of the nettle plant. In fact, patuletin has been shown to possess an antiproliferative effect on CaSki, MDA-MB-231, and SK-Lu-1 cells by activating apoptotic cell death while showing no cytotoxic effect on normal cells [64]. Additionally, the anticancer activity of caffeic acid has been demonstrated against various types of cancer, both in vitro and in vivo. Caffeic acid was reported to promote the activation of apoptosis via the extrinsic caspase-dependent pathway in melanoma cancer cells [65]. This compound was also reported for its pro-oxidant properties, along with its antiangiogenic potential and suppression of metalloproteases involved in promoting cancer cell motility and invasion [66]. In the same way, flavonoids such as quercetin and apigenin found in this extract were previously reported to exhibit an antioxidant activity in normal cells while having a pro-oxidant and pro-apoptotic effects in cancerous cells such as HCT116, HCT15, and SW480 colorectal cancer cell lines. Apigenin was also reported to exhibit its anticancer role in ovarian cancer cell lines, namely A2780, OVCAR-3, and SKOV-3 by inducing changes in ROS signaling pathways and promoting apoptosis. Moreover, caffeic acid has been described to possess an antioxidant effect in normal cells while having pro-oxidant properties in cancer cells as it enhanced lipid peroxidation in HeLa and ME-180 cervical cancer cell lines, damaging their DNA, altering the mitochondrial membrane potential, and inducing apoptosis [67]. Similarly, hydroxybenzoic acid was also investigated for its anticancer properties on cancer cells in vitro. It was previously demonstrated to promote the activation of apoptosis in breast cancer cells via the cleavage of PARP and caspase-3 proteins [68]. Other compounds found in the leaves of the nettle plant are the kaempferol and gallic acids previously evaluated for their anticancer and inhibitory effect on the migration and motility of breast, bladder, prostate, and lung cancer and hepatocellular carcinoma, among others [69,70,71]. In order to evaluate the potency of UDAE, positive controls were used, including known chemotherapeutic agents such as cisplatin. By comparing nettle tea to traditional therapies, we were able to evaluate its effectiveness and determine its potential as a complementary or alternative therapeutic option.

Collectively, the data presented in this study shed light on the promising use of nettle tea as a natural tool for chemoprevention, as demonstrated by its antiproliferative, pro-apoptotic, pro-oxidant, and antimetastatic properties on ovarian cancer cells in vitro.

## 5. Conclusions

This study is the first to examine the effect of nettle tea on aggressive ovarian cancer cells. Our data revealed the antiproliferative effect of nettle tea on SKOV-3 cells by promoting cellular fragmentation, increasing ROS production, and activating apoptosis without altering autophagy. The extrinsic apoptotic pathway was activated through the cleavage of caspases-8 and -3 along with PARP cleavage, ultimately leading to the DNA fragmentation observed in the treated cells. The inhibitory effect of UDAE on the migration and motility of ovarian cancer cells further underlines its promising therapeutic benefits. These findings emphasize the promising potential of nettle plant to be used in cancer chemoprevention and alternative medicine, especially since this paper focused on an aqueous extract, highlighting its ease and convenience of use, as well as its possibility to be integrated in the human diet as a tea. Although the potential is promising, additional studies are required to investigate and confirm the effect of UD on other ovarian cancer subtypes to understand the broad spectrum of action in vitro. Furthermore, fractionating the extract to evaluate the compounds with the highest biological therapeutic activity is of great importance. With similar importance, evaluating the extract’s effect on reactive oxygen species is critical to better understand how it regulates apoptosis, and investigating the interplay between autophagy and apoptosis, particularly in the context of ROS-dependent activation, is needed to provide further insight into its mechanism of action. Future work should aim at evaluating UD in animal models to validate the efficacy, safety, and potential side effect of using UD in living organisms, all while taking into consideration several aspects of pharmacokinetics such as bioavailability, metabolism, and tissue distribution. Finally, translating this work into clinical trial investigations is crucial for its potential implementation in cancer management or prophylaxis.

## Data Availability

The original contributions presented in the study are included in the article/Supplementary Materials, further inquiries can be directed to the corresponding authors.

## Supplementary Materials

The following supporting information can be downloaded at: https://www.mdpi.com/article/10.3390/foods13203336/s1, Table S1: List of primers used in RT-qPCR.

## Abbreviations

AML	Acute Myeloid Leukemia
DCF	2′,7′-Dichlorofluorescein
DMEM	Dulbecco’s Modified Eagle Medium
ELISA	Enzyme-Linked Immunostaining Assay
FBS	Fetal Bovine Serum
IC_50_	Half Maximal Inhibitory Concentration
LC-MS	Liquid Chromatography/Mass Spectrometry
NSCLC	Non-Small-Cell Lung Cancer
PARP	Poly-ADP Ribose Polymerase
PBS	Phosphate-Buffered Saline
PI	Propidium Iodide
PVDF	Polyvinylidene Fluoride
RIPA	Radio-Immunoprecipitation Assay
SDS-PAGE	Sodium Dodecyl-Sulfate Polyacrylamide Gel Electrophoresis
