# Supplementary material for: Protective Effects of Nettle Tea on SKOV-3 Ovarian Cancer Cells Through ROS Production, Apoptosis Induction, and Motility Inhibition Without Altering Autophagy
Source: Foods. 2024 Oct 21;13(20):3336. doi: 10.3390/foods13203336 (PMC11507475; doi:10.3390/foods13203336)
Supplement: Supplementary file 1 [file foods-13-03336-s001.zip › foods-3247545-supplementary.pdf]

Protective Effects of Nettle Tea on SKOV-3 Ovarian Cancer Cells Through ROS Production, Apoptosis Induction and Motility Inhibition Without Altering Autophagy

Supplementary Table S1: List of primers used in RT-qPCR

Gene	Forward Sequence	Reverse Sequence
A1	5′-AATTGTGCCTTGCCTGAGTGA-3′	5′-AAGAACTGCAGGTGGCTC CAT-3′
A2	5′-AATATCAACCCGGAAAAGGC-3′	5′-GTGCAACCCGTCTCGT-3′
B1	5′-CTGAGACAACTTGAGGAAGAG-3′	5’-ACCTGTACTAGCCAGTCAAT-3′
B2	5′-CAACCCACCAAAACAACA-3′	5′-AGAGCAAGGCATCAGAAA-3′
D1	5′-GAGGAGCTGCTGCAAATG-3′	5′-TTGCGGATGATCTGTTTGTT-3′
D2	5′-CTGTCACTCCTCATGACTTC-3′	5′-TGGCAAACTTAAAGTCGGTG-3′
E1	5′-GCAGGATCCAGATGAAGAA-3′	5′-TTATTGTCCCAAGGCTGGC-3′
E2	5′-TGTTGGCCACCTGTATTATCTGG-3′	5′ATCTGGAGAAATCACTTGTTCCTATTTC-3′
GAPDH	5′-AGCCTTCTCCATGGTGGTGAAGAC-3′	5′-CGGAGTCAACGGATTTGGTCG-3’
